# Classification and management of epilepsy and epileptic syndromes in a cohort of 202 school children- a 2 year follow up study- Sudan

**DOI:** 10.1186/s12883-019-1514-0

**Published:** 2019-11-15

**Authors:** Inaam N. Mohamed, Maha A. Elseed, Somia Mohamed, Ali Alsir, Emtinan K. Hamid, Ilham M. Omer, Sara M. Elsadig, Yasmin M. Gerais, Abdelgadir H. Osman, Aisha M. Bakhiet, Ahlam A. Hamed

**Affiliations:** 1 Neurology Division, Department of pediatrics and Child Health Faculty of Medicine, University of Khartoum, P. O. Box 102, Khartoum, Sudan; 2 Neurology Unit, Gafer Ibn Auf Specialized Hospital for Children, Khartoum, Sudan; 3 Neurology Unit, Soba University Hospital, Khartoum, Sudan; 4 Department of community, Faculty of Medicine, University of Khartoum, Khartoum, Sudan; 5 Department of Medicine, Faculty of Medicine, University of Khartoum, Khartoum, Sudan; 6 Ministry of Health, Khartoum, Sudan; 7 Department of Psychiatry, Faculty of Medicine, University of Khartoum, Khartoum, Sudan

**Keywords:** Classification, Management, Epilepsy, Follow up, Sudan

## Abstract

**Background:**

In this paper, seizure types, and epilepsy syndromes are elucidated as per ILAE (2010) classification. A brief outline of the antiepileptic drug regimens used and the outcome of seizure control in a two -year period is presented. The applicability of the ILAE classification in resource limited countries has been revisited.

**Methods:**

This is a descriptive prospective study, in which 202 patients were enrolled. The Cohort group was seen and evaluated by a pediatric neurologist at the Pediatric neurology Outpatients Department (OPD). Epilepsy was classified using the International League Against Epilepsy (ILAE) classification (2005–2009) report. All patients had an Electroencephalogram (EEG) at the start of the study, and this was repeated as deemed appropriate. Brain imaging (MRI) was done to patients when indicated. Treatment decisions were made by pediatric neurologists. Outcomes were categorized into four groups: fully recovered, well controlled, partially controlled and uncontrolled.

**Results:**

The mean age is 10.5 + 2.7 years. Male to female ratio was 1.7: 1. Thirty five (17.3%) patients had generalized onset seizures, 46(22.8%) had focal onset seizures, 104(51.5%) had a specific epilepsy syndrome, and 17(8.4%) patients were unclassified. 170 (84.2%) patients were on mono-therapy on their initial visit, 30(14.8%) were on two Antiepileptic Drugs (AEDs) while two (1.0%) patients were on poly-therapy. After 2 years; 155(76.7%) patients were on mono-therapy, 36(17.8%) on two AEDs while ten were (4.0%) on polytherapy. One eighty (88.2%) patients were controlled. Fifteen (7.4%) of them were off medication after being seizure free for 2 years. Twenty (9.8%) have partial control, while two (1.0%) patients were uncontrolled. Patients with focal epilepsy, those on polytherapy and those with abnormal imaging had poor prognosis.

**Conclusions:**

The ILAE classification can be used in resource limited countries. Childhood epilepsies have a good prognosis provided they are well classified and treated.

## Background

The prevalence of epilepsy is particularly high in Africa with an estimated mean prevalence of 15 per 1000 [1] The World Health Organization (WHO) estimates that there are over 50 million people with epilepsy, of whom two-thirds are children living in Africa and one-fifth in sub-Saharan Africa. The actual data upon which these estimates are made is very scarce. Epilepsy has been identified as a health priority for school age children because of its’ high psychosocial morbidity and the potential for control using low cost interventions [2].There have been relatively few studies of epilepsy in school age children in Sudan and most of these have given little information about seizure types [3] and the long-term outcome of these remains largely unknown. In this study classification of seizure types and the various epileptic syndromes according to ILAE (2010) classification was performed. This study also outlines the antiepileptic drug regimens used and the outcome of seizure freedom in a two- year period. The applicability of the ILAE classification in resource limited countries is assessed.

## Methods

This is a descriptive prospective study conducted between 2016 and 2017. The sample size calculation was based on a previous survey conducted at Khartoum State in 2015 which identified 303 children out of 74,959 school children with epilepsy and the prevalence rate was reported as 4/1000. In Phase I of this study the epilepsy was initially classified into generalized, focal and unclassified [4].

### Case definition

#### Epilepsy

A diagnosis of epilepsy in this study is retained if the patient had at least two or more epileptic seizures unprovoked by any immediate cause [1].

#### Developmental impairment

(US equivalent of mental retardation) can be static or progressive, recent or long standing, global or specific. It includes assessment of gross motor, fine motor, communication, vision, hearing, social and psychomotor skills [5].

#### Motor delay

Any delay in the acquisition of the motor skills that can easily be remembered by the parents like delayed neck control, sitting and walking [5].

#### Speech delay

Any delay in acquiring skills elated to the production and/or perception of the oral symbols used for language [5].

#### The cohort

The present study was part of a population- based study of prevalence of epilepsy in school children in Khartoum- Sudan**.** However, 50(16.5%) patients continued their follow-up in their locality or nearby clinics, 31(10.2%) had incomplete clinical data so they were excluded. Eighteen (5.9%) patients lost their follow up and we were unable to trace them while one (0.3%) patient died following a road traffic accident. In this study, we report on 202 patients whose follow up continued for 2 years.

The cohort group was seen and evaluated by the Pediatric neurologists 1&2 at the OPD at Fath El- Rahman El-Basher Center; the referred clinic for Gafer Ibn Auf Specialized Hospital for children in Khartoum- Sudan.

#### Epilepsy classification

For each patient, epilepsy was classified using the ILAE Commission on classification and terminology 2005–2009 Report [6]. Factors considered in the classification included seizure type (s) based on descriptive semiology and personal interview; however supportive information such as video recording of the event, EEG and neuroimaging findings were also utilized [2]. Patients were further classified into individual specific syndromes if applicable, based on ILAE criteria and age at onset and seizure semiology. Specific syndrome designation was assessed both at initial presentation and at prospective follow-up.

#### Investigations

All patients had an EEG at the start of the study, and that was repeated as deemed appropriate. The EEGs were done using the 10–20 system with photic stimulation and hyperventilation procedures done when indicated; none of the patients had undergone a video-telemetry EEG or an ambulatory EEG recording as these are unavailable in the study setting The majority of patients (89%) had sleep EEGs (induced by chloral-hydrate) and very few EEGs were done whilst awake or sleep deprived. The EEGs were interpreted and reported by an adult neurophysiologist with special interest in pediatrics EEGs.

The MRI was the modality of brain imaging requested as and when indicated. The MR imaging studies were performed at different centers, where the imaging protocols were variable. All images of patients in this study included at least one sagittal view and one axial sequence 1.5 T. All patients were studied with T1-T2 and FLAIR -weighted sequences. The final report was that agreed by the radiologist who finally reviewed all the images. MRI of the brain was done for patients with any impairment in development, those suspected to have behavioral and/or educational problems, those with focal seizures associated with focal neurological deficit and for patients with uncontrolled seizures.

#### Treatment

Treatment decisions were made by pediatric neurologists as per the international recommendations for the specific seizure type, taking into consideration the age of the patient, sex, tolerability, formulations, availability and affordability of AEDs in our setting. Other modalities of treatment e.g. ketogenic diet and epilepsy surgery are not available in Sudan. We tried at least two first-choice AEDs in mono-therapy (mostly Sodium Valproate and Carbamazepine) before using a polytherapy regimen. In children who experienced 2 years free of seizures, the AED(s) were withdrawn gradually.

#### Follow up and outcome

The majority of patients were seen three-monthly to check for seizures frequency, duration, drug side effects and dose adjustments of the AEDs. However, some patients were seen more frequently on an individual basis, especially if they have refractory seizures and/or other co-morbidities For the purpose of this study, the outcomes were categorized into four main groups; ‘Fully recovered’- if no seizures occurred for the last 2 years and AEDs were tapered, ‘Good’ controlled if seizures occur less than once per month. ‘Partial seizure- control’ if there is a decrease in seizure frequency and duration by more than 50% with seizures occurring every month (but not every week) during the last 6 months and lastly an ‘Uncontrolled group’ if there is no change in the seizure frequency, if it is decreased by less than 50% in the last 6 months or got worse.

#### Variables

Sex, age at onset of seizure, perinatal history, family history of epilepsy, epilepsy types, number of AEDs, MRI/ Brain findings were used as determinant to outcome.

### Data analysis

Data analysis was performed using SPSS software package version 23. The quantitative variables were presented as mean and standard deviation. Qualitative variables were described in the form of frequency and percentage. Standard test of significance (Chi-Square Test) was used. *P*-value was considered significant if its value is less than 0.05.

## Results

The study included 202 patients with epilepsy, with an age range of 6–14 years. The mean age is 10.5 + 2.7 years. The majority 101(49.5%) were more than 10 years of age. One hundred ninety (94.05%) were known to have epilepsy while 12(5.95%) were newly diagnosed. One hundred twenty six (62.4%) were male and 76(37.6%) were female with a ratio of 1.7: 1.

Forty eight (23.8%) had prenatal complications of whom 21(43.7%) suffered from Pregnancy Induced Hypertension (PIH), 15(31.3%) were affected by gestational diabetes, nine (%18.7) had complications related to Intrapartum haemorrhage and three (6.25%) were suffering from Oligohydramnios. The majority − 195(96.5%) of the study group were delivered at term, five (2.4%) were preterm and two (1.1%) were postdates. Seventeen (8.4%) patients had developmental impairment during early childhood, the majority 10(58.8%) had isolated motor impairment and seven (41.2%) had speech delay. All of them had brain atrophy on their brain images. Visual problems were reported in 12(5.9%) patients and Hearing impairment in four (1.9%) patients. Consanguinity (first degree maternal/or paternal cousin) rate was found to be 54% and 69(34.2%) patients had a positive family history of epilepsy (sibling or first degree cousin).

Thirty four (16.8%) patients had seizure onset before the age of 1 year, 49(24.1%) between 1 and 5 years, 81(40.1%) between 5 and 10 and 38(18.8%) had seizures onset after the age of 10 years.

The EEG background was normal in all patients. Generalized epileptiform discharges were recorded in the EEG of 61 (30.2%) patients, 88(43.6%) had focal seizure discharges. Fifty three (26.2%) patients had no evidence of epileptiform discharges in their first EEG of whom 20(37.7%) demonstrated evidence of epileptiform activity in the second EEG when done with sleep deprivation.

MRI of the brain was done to 118(58.4%) patients, it was abnormal in 65(55.1%), of whom 29(24.5%) had diffuse brain atrophy, 11(9.3%) had Mesial temporal sclerosis(MTS), seven (5.9%) had thin corpus callosum, five (4.2%) had evidence of infarction, four (3.3%) had congenital arachnoid cyst, four(3.3%) had hydrocephalus (with VP shunt) three(2.5%) had arrested hydrocephalus and three(2.5%) had hemi-atrophy.

One hundred eighty five (91.5%) patients had their initial epilepsy classification revised after history, clinical examination, EEG findings and the result of the MRI of the brain in addition to other investigations.

Thirty-five (17.3%) patients had generalized onset seizures, 46(22.8%) had focal onset seizures, 104(51.5%) had a specific epilepsy syndrome, and 17(8.4%) patients had unclassified seizures. Each group was further reclassified (Table 1).

**Table 1:** Classification of epilepsy among the study group (*n* = 202)

Epilepsy classification		Number (%)
Generalized 35(17.3%)	Generalized Tonic – Clonic	15(07.4)
	Myoclonic	11(05.4)
	Tonic	07(03.5)
	Atonic	02(01.0)
Focal 46(22.8%)	Simple Focal	05(02.5)
	Complex focal	15(07.4)
	Focal evolving into Generalized Seizure	26(12.9)
Epilepsy syndrome 104(51.5%)	Epilepsy with Centrotemporal Spikes(ECTS)	25(12.4)
	Childhood Absence Epilepsy (CAE)	23(11.4)
	Juvenile Absence Epilepsy (JAS)	19(09.4)
	Juvenile Myoclonic Epilepsy (JME)	15(07.4)
	Frontal lobe Epilepsy	07(03.5)
	Late-onset childhood occipital epilepsy	07(03.4)
	Epilepsy with Myoclonic Absence seizures	04(02.0)
	Hemi- convulsion-Hemiplegia-Epilepsy	03(01.5)
	Eyelid Myoclonia with Absence Seizures	01(0.5)
Unclassified 17(8.4%)	17 (8.4%)	17(08.4)
Total		202(100%)

One hundred and seventy (84.2%) patients were on mono-therapy on their initial visit, 30(14.8%) were on two AEDs while two (1.0%) patients were on poly-therapy. The most commonly used AEDS was Sodium Valproate (used by 130 (64.3%) patients) followed by Carbamazepine 87(43.0%), Phenbarbitone 19 (9.4%) and four (1.9%) patients on Topiramate.

At the initial evaluation visit 42 (20.8%) patients had good seizure control, 50(24.8%) had partial control (decrease in seizure frequency and duration by more than 50%) while 110(54.4%) had uncontrolled seizures (No change in seizure frequency or duration, or decreased by less than 50%).

After 2 years; 155(76.7%) patients were on mono-therapy, 36(17.8%) on two AEDs while ten (4.0%) on polytherapy. The commonly used AED 90(44.5%) was Sodium Valproate followed by Carbamazepine 60(29.7%), Levetiracetam 57(28.2%) and Lamotrigene in 40(19.8%) patients. Fifteen (7.4%) patients fully recovered, 165(81.7) were well controlled, twenty (9.8%) had partial control, while two (1.0%) patients were uncontrolled (Table 2).

**Table 2:** Treatment and response to AEDs at initial visit and 2- years after among the study group (*n* = 202)

	Initial Visit	After 2 years
Anti-Epileptic Drugs (AEDs)Treatment		
Mono therapy	170(84.2%)	150(76.7%)
Two drugs	30(14.8%)	36(17.8%)
polytherapy	02(1.0%)	10(4.0%)
Anti-Epileptic Drugs (AEDs)		
Sodium Valproate	130 (64.3%)	90(44.5%)
Carbamazepine	87(43.0%),	60(29.7%)
Lamotrigene	0.0	40(19.8%)
Levetiracetam	0.0	57(28.2%)
Phenbarbitone	19 (9.4%)	0.0
Topiramate	04 (1.9%)	0.0
Others	04(1.9%)	15(7.4%)
Response to AEDS		
Fully recovered	0.0	15(7.4)
Good control	42 (20.8)	165(81.7)
Partially control	50(24.7)	20(9.9)
Uncontrolled	110(54.4)	02(1.0%)
Total	202(100)	202(100)

The correlation between seizure control and other variables was tested using Chi-Square Test and *P*-value was considered significant if it is less than 0.05. There were no significant differences between males and females, age at onset of seizures, family history of epilepsy and whether there is evidence of birth asphyxia repetition (P- value = 0.1, 0.1, 0.23, 0.2) respectively.

Using Chi- Square test, patients on mono-therapy showed good seizure control when compared to those on two or more AEDs (P- value = 0.01). Patients with generalized seizures showed better control in comparison to those with focal seizures and epilepsy syndromes (*p* value = 0.002). Patients who had normal MRIBrain imaging had good seizure control and vice versa. (P- value = 0.01).

## Discussion

There is insufficient data on the prevalence of epilepsy syndromes in our population and their long term effects on the development and educational trajectories of these patients. Accurate classification of epilepsy type and specific syndromes is paramount as long-term outcome is largely determined by the underlying epilepsy syndrome. With optimal classification and management, around 70% of the patients will enter remission. In the long term, antiepileptic drugs can be discontinued in almost half of affected individuals [7]. This relatively good prognosis contrasts with persistent public stigma surrounding the condition especially in our setup [8].

Using ILAE classification (2010), we were able to classify 91.6% of epilepsies and epileptic syndromes. This was similarly reported by Eiji Oka et al. from Japan and Matti Sillanpää et al. from Finland who stated that (91%) of the patients (under 16 years) could be classified using ILAE (1989) [9, 10].

Almost half of the patients in the study had focal onset seizures a finding that is similar to the results from sub-Saharan Africa [11–14] and even a higher rate was reported from rural Kenya, in which 71% of the patients had evidence of focal onset seizures [15]. A previous report from Sudan [3] stated a higher percentage of focal-onset seizures as well. Focal seizures may still be higher in this study if more detailed investigations such as video telemetry and functional imaging (PET or SPECT) are used, which will ultimately improve the localization of epileptiform activity.

The commonest epilepsy syndrome in this study was ECTS, which was noted in 12.4% of patients. High prevalence of ECTS was also found in studies from Sweden and Italy [16–18] whereas lower frequencies were found in Israel [19]. Literature reports of frequency distribution of other epilepsies and epilepsy syndromes seem to vary considerably.

Several reports, including this study, have highlighted the relatively high frequencies of additional neurological deficits in children with epilepsy [19, 20]. In this study 8.4% patients had developmental impairment with brain atrophy on imaging. This group is likely to have a yet undiagnosed genetic/metabolic cause which is most likely the cause of both the epilepsy and the developmental impairment. Due to the lack of CGH microarray and Whole exome sequencing (WES) tests in our setting, the exact etiology in this group remains largely unknown.

Over the last decade; epidemiological studies have demonstrated that the prognosis of many childhood epilepsies is more favorable with 70–80% eventually reaching remission [21]. The prognosis of many patients in this study was good, with overall remission rates of 81.4% in comparison to a rate of 70–80% which has been reported by Tidman et al. [22]. The proportion of children who were resistant to therapy was 1% which is low when compared to Camfield et al. [23] and Elaine C et al. reports [24]. In this study, epilepsy prognosis is poor amongst patients with focal onset seizures, those on polytherapy and those with abnormal brain image when compared to those with generalized onset epilepsy and those on mono-therapy. Brorson LO et al. from Sweden reported on Long-term prognosis(12 years) in childhood epilepsy and concluded that patients with neuro-deficits had a poorer outlook [25] There was a higher probability of seizure freedom in patients receiving mono-therapy when compared to those on two AEDs or polytherapy which is similar to a report by Arts WF1 et al. [26].

Due to mounting evidence that Phenbarbitone and Topiramate can negatively impact the cognitive function in this subset of children, both were tapered and alternatives were offered [27, 28]. Alternatively, some patients were started on Levetiracetam and Lamotrigene based on recent evidence that they are the ones least likely to interfere with cognitive processes [27]. A few female patients who were initially on Sodium Valproate were shifted to Levetiracetam due to side effects mainly weight gain and hair loss and also to comply with the prevent program of avoiding Valproate in females of child bearing age.

## Conclusions

The ILAE classification can be used in resource limited countries. Childhood epilepsies have a good prognosis if well classified and treated. Further studies are needed to review long term remission, probability of recurrence after AEDs withdrawal and the impact of seizures on school performance and behavior.

## Data Availability

The dataset used and analyzed during the current study is available from the corresponding author on reasonable request.

## Abbreviations

AEDs: Antiepileptic Drugs
CAE: Childhood Absence Epilepsy
CT-Scan: Computed Tomography Scan
ECTS: Epilepsy with Centro-temporal Spikes
EEG: Electroencephalogram
JAE: Juvenile Absence Epilepsy
JME: Juvenile Myoclonic Epilepsy
MRI: Magnetic Resonance Image
WHO: World Health Organization
